# Supplementary material for: Lidar reveals pre-Hispanic low-density urbanism in the Bolivian Amazon
Source: Nature. 2022 May 25;606(7913):325–8. doi: 10.1038/s41586-022-04780-4 (PMC9177426; doi:10.1038/s41586-022-04780-4)
Supplement: Supplementary file 1 — This file contains the Supplementary Note, Supplementary Figs. 1–5, Supplementary Tables 1–5 and Supplementary Methods; see contents page for details. [file 41586_2022_4780_MOESM1_ESM.docx]

Supplementary Information for

**Lidar reveals pre-Hispanic low-density urbanism in the Bolivian Amazon**

Heiko Prümers✉, Carla Jaimes Betancourt, José Iriarte, Mark Robinson, Martin Schaich

Correspondence to: [heiko.pruemers@dainst.de](mailto:heiko.pruemers@dainst.de)

**Content**

[Supplementary Text 2](#_Toc97816504)

[Chronology of the Casarabe culture 2](#_Toc97816505)

[Funerary patterns 2](#_Toc97816506)

[Architectural elements of the civic-ceremonial architecture of the Casarabe culture. 3](#_Toc97816507)

[Description of individual monumental sites 6](#_Toc97816508)

[Supplementary Figure 1 | Orientation of earthworks and burials. 13](#_Toc97816509)

[Supplementary Figure 2 | Scatter plot. 14](#_Toc97816510)

[Supplementary Figure 3 | Radiocarbon dates from Salvatierra arranged by phase. 15](#_Toc97816511)

[Supplementary Figure 4 | Radiocarbon dates from Mendoza arranged by phase. 16](#_Toc97816512)

[Supplementary Figure 5 | Radiocarbon dates from other Casarabe culture sites. 17](#_Toc97816513)

[Supplementary Table 1 | List of Casarabe culture sites. 18](#_Toc97816514)

[Supplementary Table 2 | Radiocarbon dates from Salvatierra. 23](#_Toc97816515)

[Supplementary Table 3 | Radiocarbon dates from Mendoza. 25](#_Toc97816516)

[Supplementary Table 4 | Published radiocarbon dates from other Casarabe culture sites. 27](#_Toc97816517)

[Supplementary Table 5 | Criteria for the Monumental Mound Region settlement hierarchy. 28](#_Toc97816518)

[Supplementary Method│Oxcal code for the analysis of radiocarbon dates from Salvatierra. 29](#_Toc97816519)

# Supplementary Text

## Chronology of the Casarabe culture

For over a century, the Casarabe culture in the Llanos de Moxos has been the focus of sporadic archaeological study^46^. It is only during the last two decades that intensive archaeological excavations, survey and remote sensing have offered crucial new insights into the chronology, site construction history, subsistence and land use, and the archaeological landscape^3^. Excavations at monumental sites (locally called 'Lomas'), Salvatiera and Mendoza have revealed complex stratigraphies that bracket the chronology of the Casarabe culture from AD 500 to 1400^10-12^. The 94 radiocarbon dates from Salvatierra and Mendoza, as well as 50 additional radiocarbon dates previously published for other Casarabe culture sites^41-43, 47^, date the construction and use of these sites to this period (Supplementary Figs. 3-5, Supplementary Tables 2-4). The dates prior to this period are mostly outliers but, in some cases, also come from samples taken from the sterile soil beneath the earliest platform buildings. They date natural events or perhaps the ephemeral presence of non-ceramic cultures prior to the construction of the Casarabe culture sites. The abandonment of this system at ~CE 1400 precedes contact with Europeans and is accompanied by changes in land use with the decline of savannah burning^21^.

## Funerary patterns

Available data from Salvatierra recorded a total of 103 burials corresponding to all phases of occupation of the Casarabe Tradition. Three features are particularly noticeable: (i) the majority of the tombs lack burial goods, (ii) adults were always buried in shallow graves, and (iii) new-borns and infants were usually placed in urns characterised by large globular vessels sealed with a cover plate. Position of the body was varied (decubitus dorsal, decubitus ventral, sideways, in the foetal position, seated or kneeling), but the orientation 300^0^ W with head in the NW and feet on the SE was constant (Supplementary Fig. 1). Areas of the site as cemeteries changed over time. Some of the burials, however, received special treatment that most likely reflects their elevated status within a stratified society. The most notable of these cases is the tomb of a man between 35-40 years of age found 3 m deep in the centre of Mound 2 at Salvatierra. The body was wearing a large number of body ornaments, some of which are exotic, including a lip plug of Amazonite (likely origin Brazil), a jaguar teeth collar, three circular plates of copper, which likely formed part of his headdress and earpieces with pearls of sodalite (original from Cochabamba)^48^.

## Architectural elements of the civic-ceremonial architecture of the Casarabe culture.

*Base platforms*. A core area constituted by a human-made stepped base platform of up to 4 m in height on which the monumental buildings and smaller platforms stood. Borders of the base platforms and the axes of the platform buildings share almost the same orientation throughout the study area and are correlated with the orientation of human burials. On average, there was a deviation of 15 to 20 degrees to the west from the north-south direction. Interestingly, at some sites with two adjacent high platform buildings, one of the two has a divergent orientation (Extended Data Fig. 4b). This might reflect chronological differences, but there is no available data to analyse this pattern. There is a clear correlation between the size of the core area of sites and the height of its principal building (Supplementary Fig. 2). The average size of the core area in the 26 sites recorded by lidar, where it could be adequately determined, is 3.9 ha (a minimum of 0.5 ha and a maximum of 22.3 ha). The mean height of the civic-ceremonial architecture is 8.2 m (a minimum of 2 m and a maximum of 21 m) (Supplementary Table 1).

*Polygonal enclosures.* Polygonal enclosures that limit the settlement area and probably had a defensive function are present at 14 sites (Figs. 2-3, Extended Data Figs. 1-5, 8). At Cotoca, there are remains of several polygonal enclosures, which is probably an indication that the site grew over the centuries and that the ramparts were adapted accordingly. In its final stage, the ring wall enclosed an area of 147 ha. At Landivar, three polygonal enclosures are present, the outermost encompassing an area of 315 ha.

*Straight causeways.* Straight causeways radiating out from the core area and polygonal enclosures are present at most sites with civic-ceremonial architecture. Some of them connect sites, like those between sites 41, 106, and El Cerrito (site 33) (Extended Data Fig. 3). Others do not seem to lead to a specific place and end in the savannahs. Whether these were ceremonial pathways or paths used in daily life as a means of access to specific resources will have to be analysed by future research. At the largest sites, access to the sites might have been restricted. We interpret the numerous gaps in the causeways at their intersections with the polygonal enclosures, as well as particular platforms (about 20 m x 25 m in size and up to 2m high) located at strategic points of some of the causeways, as evidence that supports this argument (Extended Data Fig. 9b,c).

*Borrow pits.* Extensive sunken areas are directly adjacent to the core area from which earth had been taken to construct the core area platform buildings. At the largest known site, Cotoca (Figs 2, 3b, Extended Data Fig. 1), the base platform, irregular in shape and covering 22.3 ha, is 2 m above the pampas' natural levee but about 4 m above the adjacent terrain. The earth has been likely removed from a 50 to 80 m wide strip surrounding the central base platform. Today, these lower areas fill up with water during the rainy season and are swampy during much of the dry season. If there were houses located in these areas next to the settlement's core, they must have stood on stilts, or the climate must have been much drier at the time. The same is true for the second and third largest sites in the region, El Cerrito (site 33; Extended Data Fig. 3) and Landivar (site 168; Extended Data Fig. 2). At El Cerrito, earth has been removed from a 100 m wide strip to the west and north of the central platform, while at Landivar, dirt has been removed from most of the area between the central platform and the first polygonal enclosure surrounding it at a distance of 80 to 100 m.

*Reservoirs*. At first glance, it seems strange that the pre-Columbian settlers felt the need to construct water reservoirs in the Amazon lowlands. However, in the study region, as Hanagart^49^ points out, the influence of the Chaco region bordering to the south makes itself felt in lower rainfall amounts. In 2-4 months of the year, the rainfall is even <50 mm, sometimes there is no rain at all for a month. It, therefore, made sense to build water reservoirs to enable a larger population to survive in the dry season. The task was made easier by the fact that alluvial, clayey soils predominate in the region, making it difficult for rainwater to seep away. The basins therefore did not have to be made watertight by elaborate procedures. The reservoirs next to the sites with monumental architecture are all round and can be up to 40 m in diameter (Extended Data Figs. 4a, 8a,d). In some cases, they are bracketed by two semi-circular dams (Extended Data Fig. 4a). The reservoirs may have also been used for the breeding of fish and turtles^23^.

*Canals*. Previous remote sensing documented 900 km of canals showing a considerable degree of variability in form and spatial arrangement. More than half of the canals are located along the lines that connect mounds or they connect to rivers, lakes and smaller sites. They can reach several km long. Within settlement sites, canals connect to one or more reservoirs (e.g., Santa María, Extended Data Fig. 4a). Canals also connect lakes and rivers to the monumental mounds, thus integrating the settlements into the regional hydrological network. A ~4-km canal connects the topographically higher Laguna San Jose to Cotoca, which possibly brought drinking and irrigation water to the largest known monumental site in the region. In the savannahs, since the large majority of the canals do not appear to be connected to settlement sites. They are often arranged within an interconnected network of right and acute angles, ultimately draining into modern rivers. On this basis, Lombardo et al.^18^ has interpreted them as part of a system to drain the savannahs for cultivation. Canal building also entails a large amount of labour. Lombardo and Prümers^19^ calculated that on average 1-m length of canal would have involved the removal of 5m^3^ of dirt.

## Description of individual monumental sites

***Cotoca (site 185)*.** The architectural elements of the Cotoca site (Figs. 2, 3b, Extended Data Fig. 1) can be divided into three major groups: i) civic-ceremonial architecture, ii) polygonal enclosures, and iii) straight causeways radiating from the base platform.

*Platform buildings*. The centre of the Cotoca site is formed by a base platform of irregular shape that rises 4 m above the immediate surrounding ground. Because it constitutes the area where the dirt was extracted for the construction of the public architecture, it lies 0.5 m below the terrain. The base platform covers an area of about 22. 5 ha. The major concentration, greater diversity, and larger public architecture within this base platform are located in its southern sector. The underlying base platform has an oval shape in the core area. It is irregular and reaches 2-m high. A U-shaped pyramidal platform oriented to the SW is the principal building in the core area. The basal area of the pyramid is ~ 70 x 60 m, and it rises 14 m above the base platform. North of this central building are two elongated platforms (Extended Data Fig. 1, nos. 2, 3) of similar dimensions, 45 x 15 m x 2-3 m high, and oriented NNW. Another platform (no. 4), lower and oriented SW, is located on the western sector of the core area. There is another dominating platform (no. 6) of ~45 x 50 x 11 m height to the southeast U-shaped pyramidal platform. Five smaller and low platform mounds (nos.7-11) occupy the eastern half of the southern part of the core area. They are approximately square, between 20-25m wide and long, and are positioned 50-80 m apart from one another. The northern sector of the base platform is elongated and contains five platform mounds (nos. 12-16) of moderate size and height. Other platform buildings can be found on three different platforms west of the main base platform. On top of the northernmost platforms, there is a platform mound of 50 x 50 x 4.5 high (no. 19). Another impressive platform, 100 x 100 x 4 m high, is located directly north of a causeway radiating from the core area to the southwest. On top of this platform are two buildings, one of them, occupying the eastern part of the platform, is a U-shaped building (no. 17), oriented towards NNW and reaching 4 m high. The other building is a long rectangular and low platform (no.18) adjacent to the western border of the platform. South of the causeway mentioned, attached to its southwestern corner, there is another almost rectangular 1-5 m platform on top of which two low platform buildings were built. The one on the eastern end (no. 20) measures 40 x 15 m; the one on the western end (no. 21) measures approximately 20 x 10 m.

*Polygonal enclosures.* Around the core area platform, the original soil has been removed to a depth of 1m and used to construct the earthworks. Therefore, at least during the rainy season, the centre of the site was probably surrounded by water and could only be reached via causeways. In addition, the centre was protected by enclosures that were modified several times, possibly to accommodate the need for space for a larger population within the enclosed area. Northwest of the core area platform, the remains of two, only partially preserved enclosures are clearly visible. The inner one consists of a double wall with a ditch in between. The walls are still kept at the height of 1 m and are each ~ 10 m wide. The ditch between them has been heavily infilled so that today its depth is only 50 cm. The total width of this inner double-walled enclosure is considerable at 30 meters. Remains of a second, double-walled enclosure are found 50 - 80 m northwest from the enclosure mentioned above. A third enclosure, preserved in its totality, probably limited the site to its maximum extent of approximately 147 ha. In the north-western section of the site this enclosure differs from those described above in that it is constituted by a wall about 6 m wide, only about 0.5 m high, and is flanked by ditches. The latter, each about 8 m wide, are only 20-30 cm deep, but their original depth may have been two or more meters. Interestingly, the construction of the third enclosure is different in the sections southwest, south and east of the core area. There the enclosure consists of a polygonal bank and a ditch running along its outer side. A similar change in the construction probably occurred in the corresponding sections of the first and second enclosures. It is not possible to say to which of the two enclosures the remains of a low rampart between the core area platform and the third enclosure belong. In any case, they are simpler than their counterparts in the northwest of the site.

*Straight causeways.* Several raised straight causeways radiate from the central core area to the outer enclosure and from there into the surrounding country. In the enclosed space of the site, their width is mostly between 6 - 9 m, and their height reaches typically 1m. The causeways are interrupted in many places, which may be their original condition (e.g., used as screened entrances) or the result of later destruction. Notably, platforms are found where the causeways intersect with the enclosures or at points where they fork or end. Numbers 22 and 23 of Extended Data Fig. 1 represent this kind of platform, whose maximum dimensions are 25 x 30 m (see also Extended Data Fig. 9b,c).

***Landivar (*site 168).** The core area of the Landivar site (Figs. 3a, Extended Data Fig. 2) is dominated by a 5-m high, 10 ha rectangular platform, whose max dimension is 100 m long. On top of this massive structure are five platform mounds (Extended Data Fig. 2, nos. 1-5) varying in shape but sharing the same NW overall orientation. Their height ranges from 3 m (no. 5) to 5 m (no. 3). The meandering course of the paleo-river arriving at the site from the south bounds the western half of the core area of the site. It is unclear if the river was already inactive when the settlement was built or whether it dried up during the period of use of the site. Regardless, the straight raised causeways that cross the river channel in several places (nos. 6-9) show no regular water flow at the time of their construction. Even if the river was inactive, its channel likely filled with water during the rainy season and was probably waterlogged during much of the dry season. Therefore, it constituted a natural barrier that was used when constructing the inner rampart, whose course follows that of the paleo-river in it's western and north-western section. In the latter sector, there are three poorly preserved enclosures, delimiting areas of 32, 77, and 315 ha, respectively. The site has some remarkable features that, though they are also found in other sites, are only clearly shown in Landivar. These include the V-shaped causeways leading to the northern sector of the core area of the site (nos. 8-11). They may have served to restrict access to the core area, and they likely lead to the central platform through stairs. Another remarkable feature is the area bounded by a U-shaped embankment (12) to the west of the base area platform. It is a basin of about 50 x 40 m, surrounded by a horse-shoe bank, and the bottom of which is 50 cm deeper than the river channel today. The Landivar site is relatively isolated compared to the other sites in the region. However, the number of straight causeways of up to 3 km that radiate from the site show that it was also an integral part of the settlement system.

**Cerrito (site 33).** As in the primary centres, the architectural elements of the Cerrito site (Extended Data Fig. 3) are composed of platform buildings, a polygonal enclosure and straight causeways radiating from the central platform of the core. An almost quadrangular-shaped base platform located on the right bank of a paleo-river rises 6 m high and covers about 6 ha. The core area of the Cerrito site is dominated by a set of civic-ceremonial structures covering an area of almost one hectare. Although the lidar image does not allow us to determine the precise perimeter of these structures, there are remains of what may have been four structures: a rectangular building to the south ~46 x 23 x 12 m high, two buildings to the west ~18 x 20 x 8 m high and another ~37 x 20 x 8 m high, and a rectangular structure to the east ~63 x 29 x 10 m high. Opposite the northwest corner of the central architectural complex is a 30 x 13 structure. A system of at least four causeways radiates from the inner precinct in different directions, crossing the limits of an enclosed area of approximately 41 ha. A causeway in a north-easterly direction crosses an intersecting platform. There are remnants of a second enclosure to the south of the Cerrito site that delimited a much larger area but whose limits to the north are impossible to observe. In addition to its monumental architectural features, the Cerrito site is interconnected by a complex network of canals and causeways with at least eight other second and third-tier sites (Extended Data Fig. 3b).

**Santa Maria (site 193).** The layout of the Santa María site (Extended Data Fig. 4a) is much simpler than that of the Cotoca site. However, it illustrates the role that water management played in the planning in the large majority of the settlements discussed in the paper. Similar to other sites, the central sector of the settlement is adjacent to a paleo-river. Here, base platforms are found on both sides of the paleo-river, with the larger one located on the eastern side. A base platform measuring about 190 x 130 m and about 3 m high was built in this sector. On top of it stands a U-shaped platform (Extended Data Fig. 4a, no. 1) of about 6.5 m high, oriented towards the north. The base platform on the eastern side of the paleo-river is more irregular in shape and its northern sector contains three low platforms (nos. 2-4). The fact that the two platforms are connected by a dam of about 15 m wide running through the river channel shows that the paleo-river had no or very little water when the site was inhabited. A lower and narrower dam leading into the river channel directly south of the base platform from both sides indicates that water flow at the time of construction was likely minimal. However, the amount of water flowing through the paleo-river must have been sufficient to fill the two circular reservoirs located on the site. One was built directly in the old river channel (no. 5) and the other about 300 m southeast of the site's enclosure (no. 6). The enclosure (no. 7) consists of 10 m wide by 0.5 m high rampart and an outer 5-6 m wide by 0.4-0.5 m deep ditch, which was likely deeper and is now infilled.

**Salvatierra (site 108).** The detailed topographic map of Salvatierra (Extended Data Fig. 5) was the first to reveal the architectural features of the Casarabe culture sites^10, 48^. Salvatierra is located on the left bank of a paleo river. The core area is dominated by a 1.5 m high, 2 ha platform. Although the contours of its flanks are blurred, it is still discernible that the platform borders were straight. The principal building in the core area is a 7 m high platform structure (mound 1) located on the northeast side of the base platform, occupying almost a third of it. At the top of this structure is a U-shaped pyramidal platform, whose wings open to the northwest. The axis of this architectural complex shows an approximate deviation of 30º towards the west with respect to the cardinal axes. This deviation is not fortuitous; it can also be appreciated in the plan of Mound 1, as well as in all the edges of the base platform. Four smaller and low platform mounds (20x11m, 18x8m, 18x10m, 25x13m) occupy the western half of the core area. At a distance of approximately 120 meters, the centre of the mound is surrounded by a polygonal enclosure, which seems to have served a defensive function. Remains of embankments, which may have served as internal divisions and causeways, connect the core area with the enclosure. Both in the southwest and northeast of the site, the enclosure crosses the paleo-river and, in both sectors, it is not continuous exhibiting openings. However, the parts of the enclosure that restrict access are not thick enough to resist the current of a river, so it seems safe to assume that there was no or little current when the polygonal was built. Part of the rainwater that pooled in the riverbed was fed into a basin-like feature flanked by 2 m high embankments located to the north of the base platform. However, most of the water apparently was channelled into two circular basins located south of the site, each about 30 m in diameter. Motor coring of the larger reservoir closer to the settlement showed that their original surface was about 2 m below the actual surface. The capacity of the two basins can thus be calculated at about 1.400 m^3^. If we assume an average water consumption of 20 litre per capita, as determined for rural areas in the global South^50^, the basins could have supplied more than 2.000 people with water during one of the rare months without rain in the dry season^49^.

46. Nordenskiöld, E. Urnengräber und Mounds im bolivianischen Flachlande. *Baessler-Archiv* **3**, 205–255 (1913).

47. Sanematsu, K. Amazon bunmei no kenkyū = Un estudio de la civilizacion Amazonica : Kodaijin wa ikanishite shizen tono kyōsei o nashitogetanoka (Gendai Shokan, 2010).

48. Prümers, H. `Charlatanocracia´ en Mojos? Investigaciones arqueológicas en la Loma Salvatierra. Beni, Bolivia. *Boletín de Arqueología PUCP* **11**, 103-116 (2009).

49. Hannagarth, W. *Acerca de la Geoecología de las sabanas del Beni en el Noreste de Bolivia* (Instituto de Ecología, 1993).

50. U. N. D. Programme, *Beyond scarcity: Power, poverty and the global water crisis. Human Development Report 2006*, (UNDP, 2006).

#
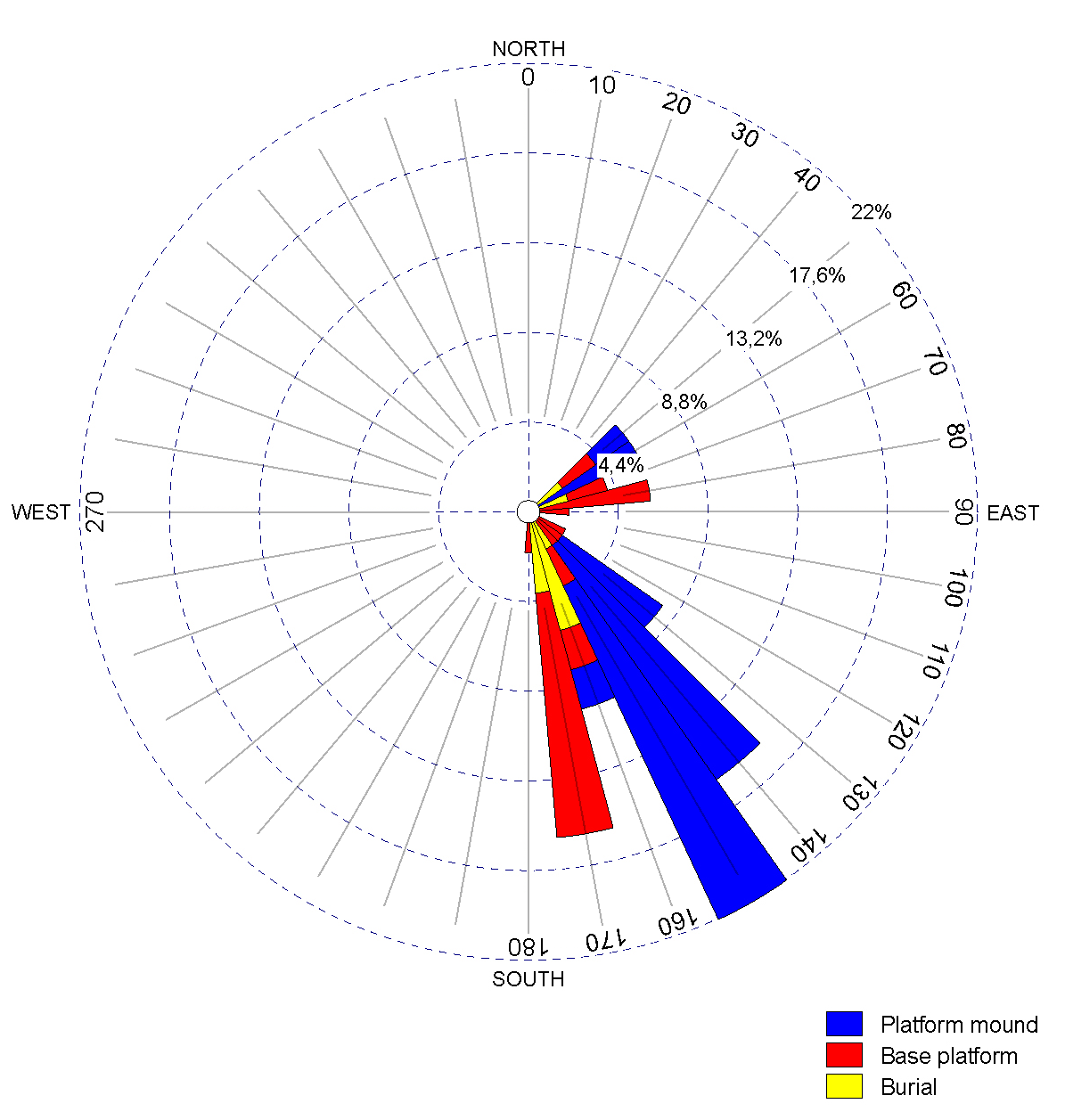


# Supplementary Figure 1 | Orientation of earthworks and burials.

The diagram shows that the predominant orientation of platform mounds, base platforms and burials is pretty much the same, mainly to SE or SSE, or 90 degrees to it.


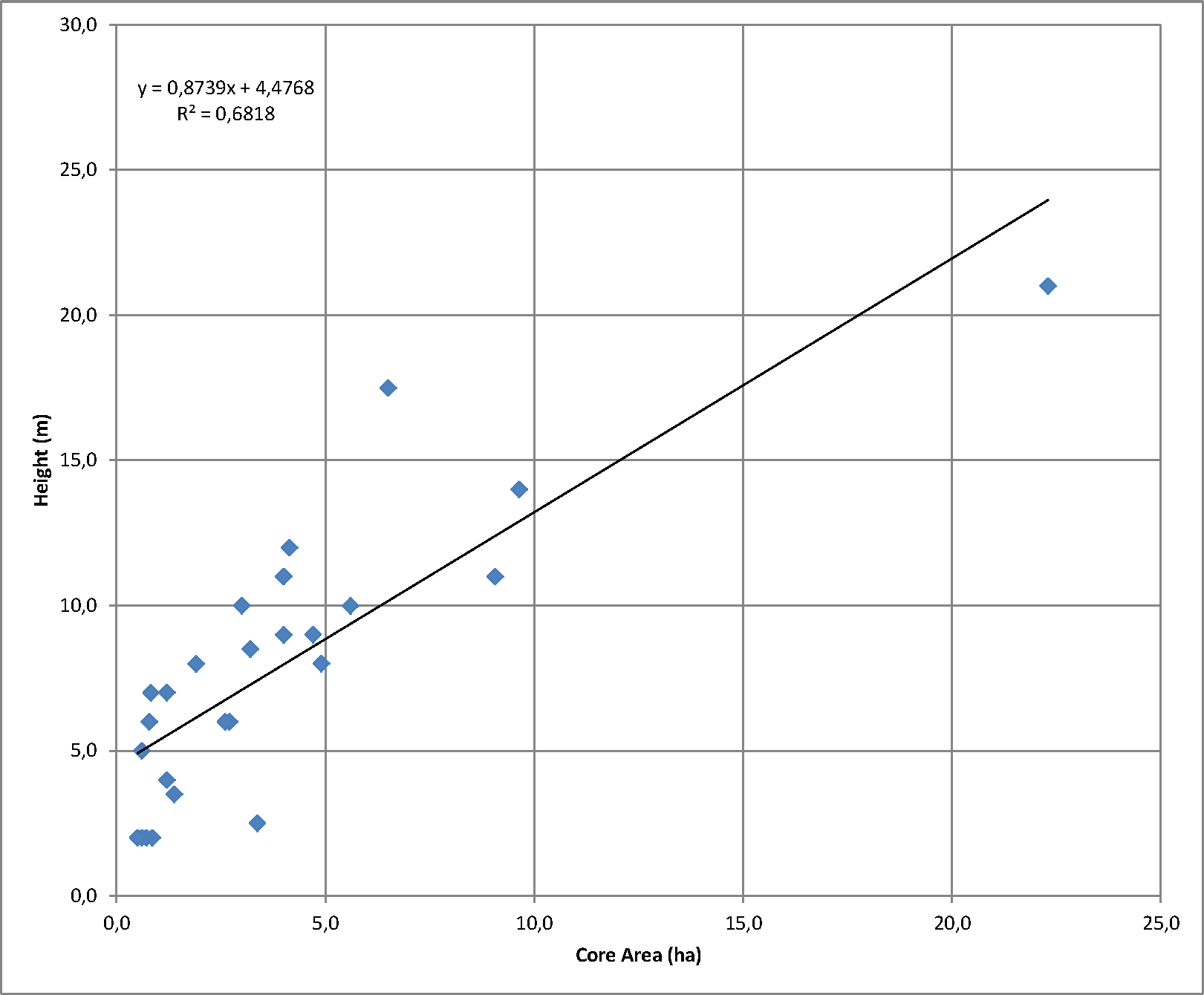


# Supplementary Figure 2 | Scatter plot.

Correlation of the height of the mayor civic-ceremonial building versus the area size of the base platform.

#
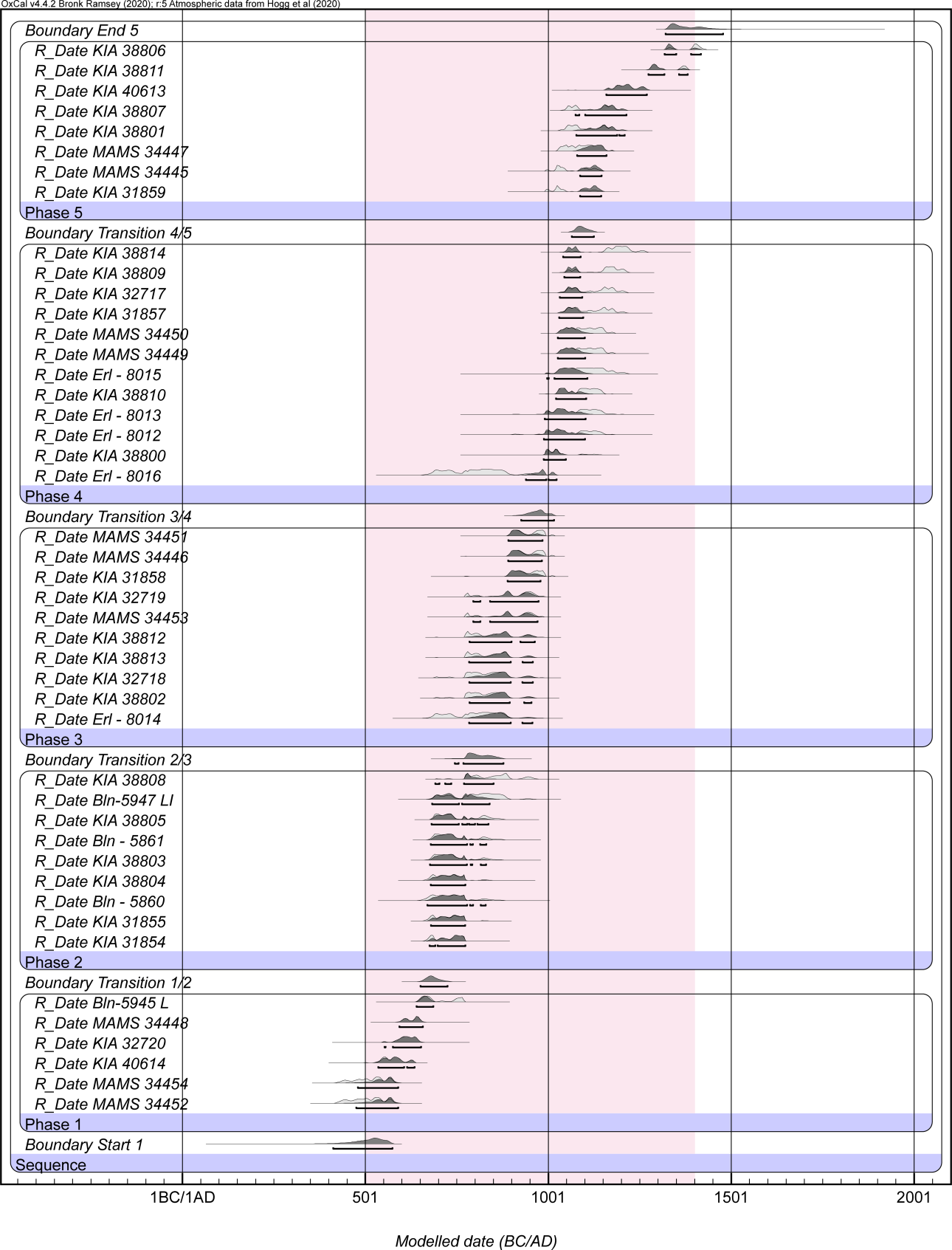


# Supplementary Figure 3 | Radiocarbon dates from Salvatierra arranged by phase.

Outliers and samples from sterile soil underlying the mound were excluded. Code is available in the Materials and Methods section.

#
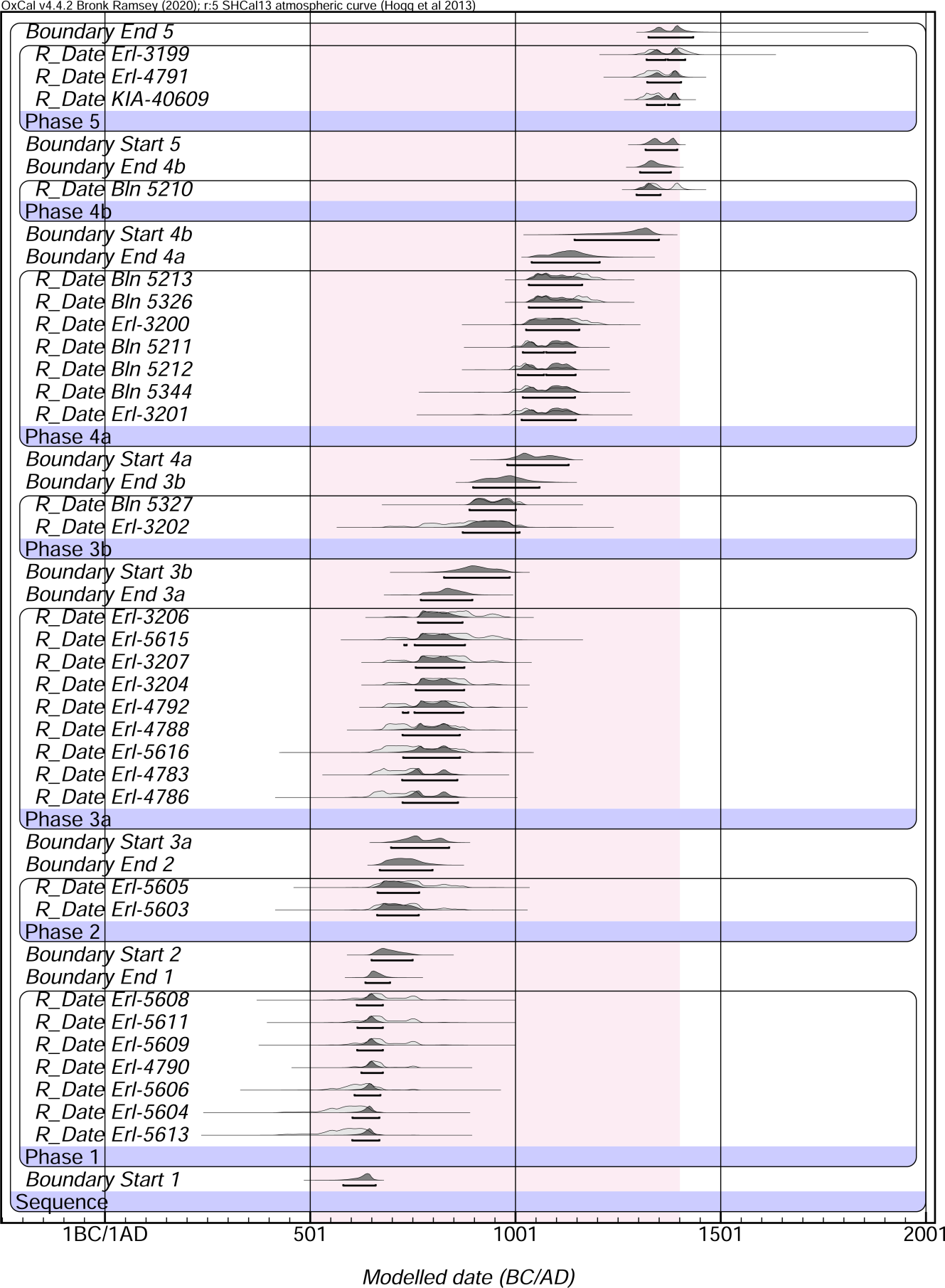


# Supplementary Figure 4 | Radiocarbon dates from Mendoza arranged by phase.

Outliers and samples from sterile soil underlying the mound were excluded^12^.

#
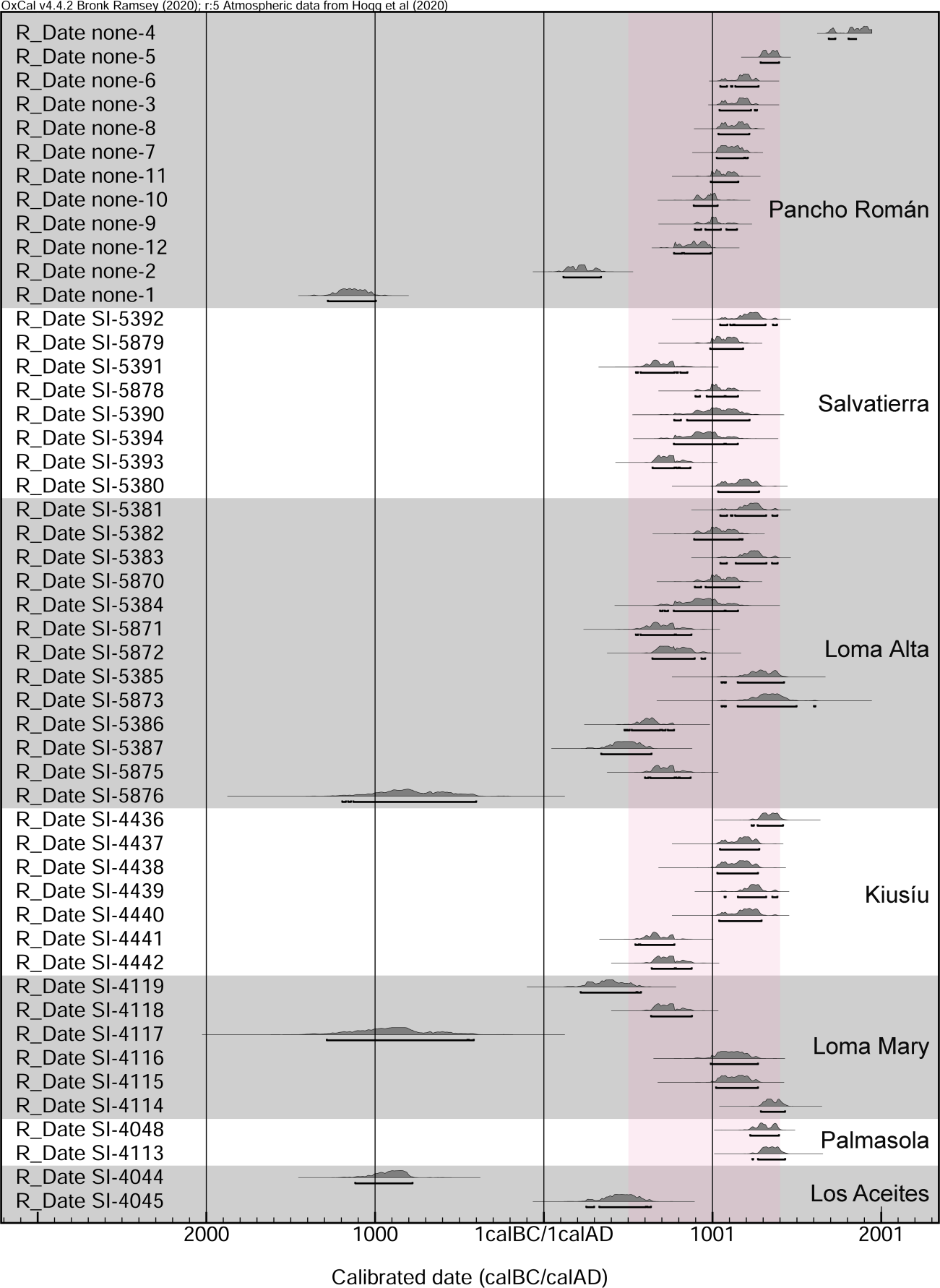


# Supplementary Figure 5 | Radiocarbon dates from other Casarabe culture sites.

These data, that had been already published^41, 43^ were recalibrated using OxCal v.4.4.2.

# Supplementary Table 1 | List of Casarabe culture sites.

| No. | Name | WGS 1984 UTM Zone 20S  x | WGS 1984 UTM Zone 20S  y | Tier | Observations |
| --- | --- | --- | --- | --- | --- |
| 1 | Lomas gemelas de la U | 339940 | 8357038 |  |  |
| 3 | Loma los guineales | 344691 | 8356979 |  |  |
| 4 | Loma de San Juaquincito | 338267 | 8350176 |  | H: 10 m |
| 5 | Irachiti 1 (lomita) | 340692 | 8362187 |  |  |
| 6 | Loma de los Tacuarales | 346716 | 8368995 |  |  |
| 7 | Irachiti 2 (Loma grande) | 340503 | 8363314 |  |  |
| 8 | Los Cafeces | 337924 | 8363125 |  |  |
| 9 | Quillisa 1 | 342310 | 8360379 |  |  |
| 10 | Tacuarita | 342753 | 8365506 |  |  |
| 11 | Tatoyeque | 347952 | 8365827 |  |  |
| 12 | Bali 1 | 354012 | 8367627 |  |  |
| 13 | Bali 2 | 353894 | 8367839 |  |  |
| 14 | Trapiche | 345733 | 8364812 |  |  |
| 16 | Ibibate | 348470 | 8362799 | 2 | H: 20 m |
| 17 | Santa Fe | 351012 | 8360933 |  |  |
| 18 | Sinaí | 351850 | 8360631 |  |  |
| 19 | Mururé-urure | 351903 | 8361739 |  |  |
| 20 | Santa Elena | 355264 | 8358575 |  |  |
| 21 | Lomita de Benjamin 3 | 355981 | 8356408 |  |  |
| 22 | Loma de Benjamin 1 | 356159 | 8356333 |  |  |
| 23 | Loma de la poza de lo Hochoose | 354198 | 8357302 |  |  |
| 24 | Loma de ida a los portones | 347792 | 8356755 |  |  |
| 25 | Loma de la U - Kia aa | 352776 | 8356111 |  |  |
| 26 | Tambata | 355131 | 8363563 | 2 | H: 15 m |
| 27 | Tutumo 1 | 353582 | 8353587 |  |  |
| 28 | Tutumo 2 | 353711 | 8353398 |  | H: 5 m |
| 29 | Benjamin 2 | 356100 | 8356601 |  |  |
| 30 | Isla de monte | 351031 | 8357023 |  |  |
| 31 | Los portones | 350407 | 8356070 |  |  |
| 32 | Loma 1 | 331623 | 8360958 |  |  |
| 33 | Cerrito | 328998 | 8363198 | 2 | H: 16 m; core area 6,52 ha; max. area: 47,3 ha |
| 36 | Cerrito - Loma del puente | 329519 | 8362294 |  |  |
| 37 | Loma 3 | 333220 | 8361490 |  |  |
| 38 | Loma 4 | 332926 | 8360683 |  |  |
| *39 | Lidar Area Cerrito - mound 3 | 330901 | 8359790 | 3 | H: 11 m; core area: 3,9 ha |
| *40 | Lidar Area Cerrito - mound 40 | 325637 | 8343053 | 3 | H: 4 m; corea area: 1,2 ha |
| *41 | Lidar Area Cerrito - mound 41 | 323462 | 8358856 | 3 | H: 7 m; core area: 3,2 ha |
| *42 | Lidar Area Cerrito - mound 42 | 324523 | 8363355 | 3 | core area: 4,9 ha |
| 43 | Loma de Fernando Velasco | 319451 | 8364845 |  | H: 4 m |
| 44 | -/- | 321903 | 8367953 |  |  |
| 45 | Estancia Las Pavas 1 | 317057 | 8377209 |  |  |
| 46 | Estancia Las Pavas 3 | 320101 | 8382364 |  |  |
| 47 | Estancia Las Pavas 2 | 316163 | 8388881 |  |  |
| *48 | Lidar San Nicolas, Site 3 | 327306 | 8396162 | 3 | H: 3 m; core area: 0,8 ha; max. area: 4,7 ha |
| *49 | Lidar San Nicolas, Site 2 | 326749 | 8398366 | 3 | H: 2 m; core area: 1 ha |
| *51 | Lidar San Nicolas, site 1, mound 2 | 327194 | 8399796 | 2 | H: 17 m; core area: 9,7 ha |
| *53 | Lidar La Punta, site 2, mound 2 | 327208 | 8404857 | 2 | H: 10 m; core area: 5,6 ha |
| *54 | Lidar La Punta, Sitio 3 | 328025 | 8404710 | 2 | core area: 2,7 ha |
| 55 | La Punta | 324528 | 8397381 | 3 | H: 9 m; core area: 1,9 ha |
| 57 | Berdun - Narasaquige | 294196 | 8390775 |  |  |
| 58 | Tegere | 297339 | 8390281 |  |  |
| 59 | La Loma | 299927 | 8389533 |  |  |
| 60 | Loma Pecine | 292979 | 8389440 |  | H: 4 m |
| 61 | Loma Tanaka | 303818 | 8386791 | 3 | H: 5 m; max. area 6,1 ha |
| 62 | Tegere | 295332 | 8385066 |  | H: 4 m |
| 63 | Espíritu Santo | 304402 | 8381943 |  |  |
| 64 | Cernández | 307414 | 8377608 |  |  |
| 65 | Loma Laguna Cernández | 309441 | 8375592 |  | H: 4 m |
| 66 | Berdun-Narasaquige | 290380 | 8386487 |  | H: 3 m |
| 67 | El pesine | 289106 | 8385572 |  | H: 4 m |
| 68 | Loma de Juanicuchi | 289729 | 8383175 |  |  |
| 69 | Loma Jocreni | 288420 | 8379513 |  |  |
| 70 | Loma Coimbra | 292640 | 8371677 |  |  |
| 71 | Chuchini | 290458 | 8371062 |  |  |
| 72 | Naranjito | 292859 | 8369113 |  |  |
| 73 | Loma Suarez | 288695 | 8366972 |  |  |
| 74 | Muñoz | 288228 | 8365718 |  | H: 5 m |
| 75 | Loma de Puerto Ballivián | 286925 | 8362801 |  | H: 4 m |
| 76 | Los Tojos / Catalayut | 287200 | 8362407 |  | H: 4 m |
| 77 | -/- | 286875 | 8359240 |  |  |
| 78 | la Bomba | 288007 | 8357936 |  |  |
| 79 | Loma Santa | 288401 | 8357786 |  |  |
| 80 | Jalisco | 288543 | 8357153 |  | H: 2 m |
| 81 | Cachipere | 285655 | 8355034 |  | H: 4 m |
| 82 | Cachipere | 286033 | 8354483 |  |  |
| 83 | Loma de la Santíssima Trinidad | 295094 | 8358987 |  |  |
| *84 | Area Cerrito - Mound 4 | 328175 | 8360998 | 3 | H: 5 m; core area: 1 ha |
| 85 | Lomita de la nueva trinidad | 297953 | 8361048 |  | H: 1 m |
| 86 | Colegio Fe y Alegría | 299129 | 8360764 |  | H: 1 m |
| 87 | Lomita Cotoca | 299140 | 8361811 |  |  |
| 89 | General Vaca Medrano | 300932 | 8361485 |  |  |
| 90 | Los Tacuaritas | 297398 | 8355905 |  |  |
| 91 | Loma del Dr. Skandar | 295646 | 8352570 |  |  |
| 92 | Palmasolo | 288715 | 8352578 |  |  |
| 93 | Perro Muerto 1 | 305929 | 8350214 |  |  |
| 94 | Perro Muerto 2 | 306509 | 8351589 |  |  |
| 95 | Los Curupauces | 304260 | 8369741 |  |  |
| 96 | Los Sumuqueces 1 | 305324 | 8367848 |  |  |
| 97 | Los Sumuqueces 2 | 306206 | 8366998 |  | H: 3 m |
| 98 | Loma de Huberto Torrico | 305639 | 8363228 | 3 | H: 5 m; core area: 1,3 ha |
| 99 | Chichi Gómez | 305450 | 8362690 |  |  |
| 100 | Campamento - Mound 1 | 306605 | 8359823 |  |  |
| 101 | Los Curupauces | 308062 | 8369362 |  | H: 2 m |
| 102 | Laguna Cernández 2 | 309424 | 8368261 |  |  |
| 103 | El Chaco 1 | 309608 | 8355406 |  |  |
| 104 | El Chaco 2 | 310621 | 8353675 |  | H: 2 m |
| 105 | La Chacra | 314261 | 8355647 |  |  |
| 106 | Area Cerrito - Mound 3 | 327113 | 8357940 | 3 | H: 5 m; core area: 2,6 ha; max. area: 15,9 ha |
| 107 | Pancho Román | 328799 | 8350032 |  |  |
| 108 | Salvatierra | 340133 | 8354024 | 2 | H: 9 m; core area: 4,0 ha; max. area: 21,1 ha |
| 109 | Población de Casarabe | 341110 | 8355066 |  |  |
| 110 | Loma Alta de Casarabe | 341495 | 8355493 |  |  |
| 111 | Víbora | 342448 | 8357908 |  |  |
| 112 | Ibiato | 344164 | 8359702 |  |  |
| 113 | Didier | 342404 | 8363750 |  |  |
| 114 | del Tigre | 351531 | 8380105 |  |  |
| 115 | Kenia | 354426 | 8376798 |  |  |
| 116 | Dulcita | 358778 | 8379326 |  |  |
| 117 | San Silvestre | 359274 | 8379131 |  |  |
| 119 | Viña Florida | 346400 | 8350389 |  |  |
| 120 | Villa Caterine | 346311 | 8350376 |  |  |
| 121 | -/- | 338554 | 8348904 |  |  |
| 122 | 19 de Junio | 346224 | 8346286 |  |  |
| 123 | Curva de Villa Banzer | 346848 | 8345730 |  |  |
| 124 | Villa Banzer | 348572 | 8346877 |  |  |
| 125 | Loma de Balvino Pardo | 349395 | 8347776 |  | H: 6 m |
| 126 | 19 de Junio / 76 | 351444 | 8346527 | 2 | H: 10 m |
| 127 | Loma de don Antonio Vaca | 354131 | 8348022 |  | H: 5 m |
| 128 | Loma de la Laguna El Tamarindo | 356006 | 8345774 |  | H: 4 m |
| 129 | Loma de Carlos Nosa Teco | 359023 | 8345429 |  | H: 6 m |
| 130 | Loma de Desiderio Nosa | 357072 | 8343857 |  | H: 4 m |
| 131 | Ave María | 364864 | 8344590 |  |  |
| 132 | Guajojó | 364027 | 8340938 |  |  |
| 133 | Latorre | 363538 | 8340484 |  |  |
| 134 | Loma del coregidor | 359691 | 8340257 |  | H: 4 m |
| 135 | Loma de Leonor Chávez | 356866 | 8342104 |  |  |
| 136 | de Juan Carlos Fernández | 355271 | 8341326 |  |  |
| 137 | Loma del Carmen | 354190 | 8342211 |  |  |
| 138 | Buen Jesús | 352873 | 8342664 |  |  |
| 139 | Estrella de Belén | 354373 | 8341597 |  |  |
| 140 | Loma 5 | 354374 | 8341477 |  |  |
| 141 | Tokio | 352796 | 8340573 |  |  |
| 142 | de Dante Cosio | 351961 | 8340322 |  |  |
| 143 | de Manuela Herrera | 352144 | 8339801 |  |  |
| 144 | Simeón Negrete | 350944 | 8340562 |  |  |
| 145 | Santo Domingo | 347800 | 8340092 |  |  |
| 146 | El Cuarto del Doctor Sergio Ortiz | 355082 | 8337975 |  |  |
| 147 | de Wilfredo Vaca Martínez | 362127 | 8337656 |  |  |
| 148 | Carina Elita | 358921 | 8335325 |  |  |
| 149 | Valladolid | 358775 | 8334771 |  |  |
| 150 | Poza Honda | 362939 | 8334325 |  |  |
| 151 | Pero Achéau | 356693 | 8333221 |  | H: 5 m |
| 152 | -/- | 356037 | 8333218 |  |  |
| 153 | de José Luis Peña | 354030 | 8334189 |  |  |
| 154 | La Asunta | 347651 | 8331295 |  | H: 6 m |
| 155 | -/- | 346712 | 8330777 |  |  |
| 156 | Loma de San Marcos - La Victoria | 350237 | 8325881 |  | H: 6 m |
| 157 | Loma de Luis Román | 354964 | 8328877 |  | H: 3 m |
| 158 | Loma Rica | 359279 | 8330348 |  |  |
| 159 | Vistosa | 362027 | 8330088 |  |  |
| 160 | San Juan de Mocobí | 357350 | 8327507 |  |  |
| 161 | Soledad | 368628 | 8329665 |  |  |
| 162 | Solano | 372334 | 8328762 |  |  |
| 162 | Bei | 372334 | 8328762 |  |  |
| 163 | Mur - 1 | 378633 | 8328919 |  | H: 6 m |
| 164 | Mur - 2 | 385943 | 8323516 |  |  |
| 165 | Escuela Caimanes | 386928 | 8323583 |  |  |
| 166 | El Tacuaral | 386595 | 8322580 |  |  |
| 167 | -/- | 354220 | 8312018 |  |  |
| 168 | Landivar | 342107 | 8318852 | 1 | H: 10 m; core area: 11 ha; max. area: 314,8 ha |
| 174 | Chocolatal - 2 | 319796 | 8331949 |  | H: 5 m |
| 175 | Chocolatal - 1 | 319558 | 8332865 |  |  |
| 176 | El Coquino | 319942 | 8335922 |  | H: 1 m |
| 177 | Chichi Munguía | 315198 | 8336599 |  | H: 4 m |
| 178 | Córdoba | 310557 | 8339318 |  |  |
| 179 | -/- | 313200 | 8338192 |  |  |
| 180 | San Antonio | 315738 | 8339769 |  |  |
| 181 | Belleza | 316022 | 8340594 |  |  |
| 182 | Aldo Monasterio | 320980 | 8338821 |  |  |
| 183 | -/- | 322312 | 8338716 |  |  |
| 185 | Cotoca | 328673 | 8342813 | 1 | H: 20 m; core area: 22,3 ha; max. area: 147,2 ha |
| 186 | Sirari (Chocolatalito) | 324716 | 8343896 | 2 | H: 10 m; core area: 3,1 ha; max. area: 26,8 ha |
| 187 | Samuray | 324559 | 8344672 | 3 | H: 3 m; core area: 1,3 ha |
| *189 | Lidar Area Cotoca - s.n. 1 | 322202 | 8346234 | 4 | H: 2 m; core area: 0,25 ha; max. area: 2,3 ha |
| 190 | Somopae | 321321 | 8347058 |  |  |
| 191 | Miraflores | 320600 | 8346924 |  |  |
| *192 | Loma de Juan Rivera | 320080 | 8346298 | 4 | H: 2 m; core area: 0,28 ha; max. area: 2,5 ha |
| 193 | Santa María | 317270 | 8345209 | 2 | H: 7 m; core area: 4,7 ha; max. area: 26,7 ha |
| 194 | Mendoza | 343929 | 8354012 |  |  |
| *195 | Lidar Area Cotoca - Mound 195 | 320698 | 8342798 | 3 | H: 5 m; core area: 1,2 ha ; max. area: 5,1 ha |
| 196 | Puesto militar de Casarabe | 346002 | 8352784 |  |  |
| 197 | El Cerrito | 301377 | 8366539 |  | H: 4 m |
| 198 | -/- | 294153 | 8346722 |  |  |
| 199 | Trinidad Viejo | 288214 | 8347202 |  |  |
| 209 | Campamento - Mound 2 | 306823 | 8359895 |  |  |
| 211 | Loma Colegio | 310656 | 8340075 |  |  |
| 213 | Loma del gran cacique | 361359 | 8341551 |  | H: 6 m |
| 301 | Mari | 281137 | 8352695 |  |  |
| 302 | Kiusiu | 279766 | 8353847 |  |  |

Coordinates are UTM 20 S WSG84

* unknown prior to Lidar-mapping.

# Supplementary Table 2 | Radiocarbon dates from Salvatierra.

| No. | Lab. code | Provenience | Material | Phase | Date BP | unmodelled (95.4%) | modelled (95.4%) |
| --- | --- | --- | --- | --- | --- | --- | --- |
| 1* | Bln-5946 LI | Unit 2, Feature 2133, cuadr. E/1, 370-380 cm | Charcoal | pre | 3319±42 | 1676-1439 BC | 1640-1427 BC |
| 2** | KIA 31856 | Unit 1, Feature 11, grave | Bone | 1 | 1623±30 | 413-574 AD | 496-596 AD |
| 3 | MAMS 34452 | Unit 2, Feature 2051, cuadr. D/5, 312 cm | Charcoal | 1 | 1594±23 | 429-578 AD | 495-591 AD |
| 4 | MAMS 34454 | Unit 10, Feature 1134, cuadr. 203/112, 200-210 cm | Charcoal | 1 | 1589±22 | 432-580 AD | 504-597 AD |
| 5 | KIA 40614 | Unit 1, Feature 1051, cuadr. 208/105, 290-300 cm | Charcoal | 1 | 1542±21 | 532-636 AD | 541-636 AD |
| 6 | KIA 32720 | Unit 10, Feature 1153, cuadr. 207/112, 270-280 cm | Charcoal | 1 | 1489±27 | 552-652 AD | 577-652 AD |
| 7 | MAMS 34448 | Unit 1, Feature 1042, cuadr. 203/102, 230-240 cm | Charcoal | 1 | 1464±22 | 594-658 AD | 595-658 AD |
| 8 | Bln-5945 L | Unit 2, Feature 2123, cuadr. E/8, 330-340 cm | Charcoal | 1 | 1389±30 | 641-774 AD | 639-680 AD |
| 9 | KIA 31854 | Unit 1, Feature 1001, grave | Bone | 2 | 1350±20 | 669-774 AD | 670-757 AD |
| 10 | KIA 31855 | Unit 1, Feature 1005, grave | Bone | 2 | 1341±24 | 671-774 AD | 672-755 AD |
| 11 | Bln - 5860 | Unit 2, Feature 2050, cuadr. 1-2/D, 260-270 cm | Charcoal | 2 | 1337±39 | 654-853 AD | 670-755 AD |
| 12 | KIA 38804 | Unit 9, Feature 921, grave | Bone | 2 | 1335±26 | 666-826 AD | 673-753 AD |
| 13 | KIA 38803 | Unit 9, Feature 912, grave | Bone | 2 | 1316±27 | 673-852 AD | 678-749 AD |
| 14 | Bln - 5861 | Unit 5, Feature 5022, cuadr. 213-214/102-103, 280-300 cm | Charcoal | 2 | 1311±26 | 676-860 AD | 680-747 AD |
| 15 | KIA 38805 | Unit , Feature 925, grave | Bone | 2 | 1300±23 | 681-870 AD | 683-744 AD |
| 16 | Bln-5947 LI | Unit 10, Feature 1142, cuadr. 203/111, 220-230 cm | Charcoal | 2 | 1275±39 | 681-891 AD | 680-746 AD |
| 17 | KIA 38808 | Unit 2, Feature 2059, grave | Bone | 2 | 1224±24 | 772-961 AD | 690-738 AD |
| 18** | Erl - 8011 | Unit 1, Feature 17A, cuadr. 208/101-102, 130-140 cm | Charcoal | 3 | 2594±49 | 817-486 BC | 817-486 BC |
| 19 | Erl - 8014 | Unit 2, Feature 227, cuadr. 5/E, 190-200 cm | Charcoal | 3 | 1275±46 | 678-952 AD | 733-957 AD |
| 20 | KIA 38802 | Unit 2, Feature 236, grave | Bone | 3 | 1240±28 | 694-959 AD | 771-953 AD |
| 21 | KIA 32718 | Unit 4, Feature 4130, cuadr. E/12, 370 - 380 cm | Charcoal | 3 | 1239±31 | 692-963 AD | 771-957 AD |
| 22 | KIA 38813 | Unit 6, Feature 6007, grave | Bone | 3 | 1228±23 | 772-957 AD | 772-957 AD |
| 23 | KIA 38812 | Unit 6, Feature 6005, grave | Bone | 3 | 1223±26 | 772-963 AD | 772-962 AD |
| 24 | MAMS 34453 | Unit 4, Feature 4084, cuadr. E/17, 350-360 cm | Charcoal | 3 | 1204±23 | 771-986 AD | 772-975 AD |
| 25 | KIA 32719 | Unit 4, Feature 4148, cuadr. B/19, 390-400 cm | Charcoal | 3 | 1201±24 | 772-987 AD | 772-984 AD |
| 26 | KIA 31858 | Unit 4, Feature 4098, grave | Bone | 3 | 1156±25 | 891-992 AD | 891-991 AD |
| 27 | MAMS 34446 | Unit 2, Feature 217, cuadr. 3/D, 175,5 cm | Charcoal | 3 | 1149±22 | 893-993 AD | 893-992 AD |
| 28 | MAMS 34451 | Unit 4, Feature 4091, cuadr. A/19, 320-330 cm | Charcoal | 3 | 1145±22 | 893-994 AD | 893-993 AD |
| 29 | Erl - 8016 | Unit 4, Feature 437, cuadr. 17/A, 210-220 cm | Charcoal | 4 | 1278±53 | 673-958 AD | 978-1030 AD |
| 30 | KIA 38800 | Unit 1, Feature 18a, grave | Bone | 4 | 1060±25 | 989-1137 AD | 994-1048 AD |
| 31 | Erl - 8012 | Unit 2, Feature 205, cuadr. 3/C, 150 cm | Charcoal | 4 | 1044±44 | 909-1157 AD | 998-1088 AD |
| 32 | Erl - 8013 | Unit 2, Feature 214, cuadr. 6/D, 140,8 cm | Charcoal | 4 | 1030±44 | 990-1159 AD | 1000-1090 AD |
| 33 | KIA 38810 | Unit 4, Feature 4096, grave | Bone | 4 | 1007±22 | 1025-1150 AD | 1022-1090 AD |
| 34 | Erl - 8015 | Unit 4, Feature 446, cuadr. 20/E, 200 cm | Charcoal | 4 | 985±45 | 1020-1210 AD | 1020-1090 AD |
| 35 | MAMS 34449 | Unit 4, Feature 4018, cuadr. C/19, 240-250 cm | Charcoal | 4 | 982±23 | 1030-1157 AD | 1029-1087 AD |
| 36 | MAMS 34450 | Unit 4, Feature 4061, cuadr. D/15, 280-290 cm | Charcoal | 4 | 981±22 | 1030-1157 AD | 1030-1087 AD |
| 37 | KIA 31857 | Unit 1, Feature 12, grave | Bone | 4 | 956±25 | 1036-1207 AD | 1035-1086 AD |
| 38 | KIA 32717 | Unit 4, Feature 4085 / 4091, cuadr. A/20, 310-320 cm | Charcoal | 4 | 948±25 | 1045-1212 AD | 1038-1087 AD |
| 39 | KIA 38809 | Unit 2, Feature 2103, grave | Bone | 4 | 932±22 | 1047-1219 AD | 1045-1085 AD |
| 40 | KIA 38814 | Unit 1, Feature 19, grave | Bone | 4 | 896±32 | 1052-1270 AD | 1045-1085 AD |
| 41 | KIA 31859 | Unit 5, Feature 506, grave | Bone | 5 | 1041±20 | 991-1145 AD | 1085-1145 AD |
| 42 | MAMS 34445 | Unit 2, Feature 202, cuadr. 3/E, 120 cm | Charcoal | 5 | 1033±21 | 992-1148 AD | 1085-1145 AD |
| 43 | MAMS 34447 | Unit 3, Feature 301, cuadr. 8/B, 7 cm | Charcoal | 5 | 988±22 | 1030-1154 AD | 1081-1158 AD |
| 44 | KIA 38801 | Unit 1, Feature 97, grave | Bone | 5 | 957±23 | 1035-1185 AD | 1078-1207 AD |
| 45 | KIA 38807 | Unit 12, Feature 1219, grave | Bone | 5 | 947±21 | 1045-1213 AD | 1078-1213 AD |
| 46 | KIA 40613 | Unit 4, Feature 424, cuadr. 19/E, 148 cm | Charcoal | 5 | 876±29 | 1158-1271 AD | 1159-1270 AD |
| 47 | KIA 38811 | Unit 4, Feature 4209, grave | Bone | 5 | 740±23 | 1275-1385 AD | 1273-1380 AD |
| 48 | KIA 38806 | Unit 12, Feature 1208, grave | Bone | 5 | 596±22 | 1324-1426 AD | 1317-1414 AD |

* from sterile soil

** outlier

# Supplementary Table 3 | Radiocarbon dates from Mendoza.

| No. | Lab. no. | Provenience | Material | Phase | Date BP | cal (95.4%) |
| --- | --- | --- | --- | --- | --- | --- |
| 1 | Bln 5328 | Unit 1, Feature 48, cuadr. 25/A, 550 - 560 cm | Charcoal | - | 2800±29 | 1000 - 820 BC |
| 2 | Erl-5613 | Unit 6, Feature 638, cuadr. 55/A, 490-500 cm | Charcoal | 1 | 1512±54 | 440 - 670 AD |
| 3 | Erl-5611 | Unit 6, Feature 619, cuadr. 57/B, 460-470 cm | Charcoal | 1 | 1407±52 | 580 - 780 AD |
| 4 | Erl-5608 | Unit 5, Feature 569, cuadr. 61/B, 500-510 cm | Charcoal | 1 | 1407±55 | 570 - 840 AD |
| 5 | Erl-5612 | Unit 6, Feature 629, cuadr. 59/A, 480-490 cm | Charcoal | 1 | 1313±52 | 660 - 890 AD |
| 6 | Erl-4790 | Unit 5, Feature 540, cuadr. 61/A, 410 cm | Charcoal | 1 | 1417±36 | 590 - 770 AD |
| 7 | Erl-5610 | Unit 6, Feature 606, cuadr. 59/A, 380-390 cm | Charcoal | 1 | 2244±54 | 400 - 110 BC |
| 8 | Erl-5609 | Unit 6, Feature 533, cuadr. 57/B, 370 cm | Charcoal | 1 | 1410±53 | 580 - 780 AD |
| 9 | Erl-5604 | Unit 5, Feature 542, cuadr. 62/B, 440-450 cm | Charcoal | 1 | 1508±51 | 470 - 670 AD |
| 10 | Erl-5607 | Unit 5, Feature 558, cuadr. 65/B, 490 cm | Soot | 1 | 1342±60 | 640 - 880 AD |
| 11 | Erl-5606 | Unit 5, Feature 558, cuadr. 65/A, 480-490 cm | Charcoal | 1 | 1465±53 | 520 - 770 AD |
| 12 | Erl-5603 | Unit 5, Feature 515 A, cuadr. 65/A, 425 cm | Charcoal | 2 | 1357±51 | 640 - 860 AD |
| 13 | Erl-3205 | Unit 5, Feature 515, cuadr. 61/A, 290-300 cm | Charcoal | 2 | 1253±36 | 680 - 960 AD |
| 14 | Erl-5614 | Unit 7, Feature 704, cuadr. 54/A, 170-180 cm | Charcoal | 2 | 1235±53 | 680 - 980 AD |
| 15 | Erl-5605 | Unit 5, Feature 547, cuadr. 69/A, 420-430 cm | Charcoal | 2 | 1338±51 | 650 - 870 AD |
| 16 | Erl-4785 | Unit 3, Feature 328, cuadr. 27/107-108, 170-175 cm | Charcoal | 2 | 1489±54 | 470 - 680 AD |
| 17 | Erl-4789 | Unit 5, Feature 512, cuadr. 63/A, 300-305 cm | Charcoal | 3a | 1557±39 | 430 - 640 AD |
| 18 | Erl-4788 | Unit 5, Feature 511 A, cuadr. 64/A, 320-330 cm | Charcoal | 3a | 1296±36 | 680 - 880 AD |
| 19 | Erl-3206 | Unit 5, Feature 509, cuadr. 65/A, 310-320 cm | Charcoal | 3a | 1231±38 | 690 - 980 AD |
| 20 | Erl-4787 | Unit 5, Feature 507, cuadr. 66/A, 340 cm | Charcoal | 3a | 1443±38 | 580 - 760 AD |
| 21 | Erl-4786 | Unit 5, Feature 502, cuadr. 67/A, 274 cm | Charcoal | 3a | 1371±49 | 630 - 860 AD |
| 22 | Erl-3207 | sector E, Feature 501, cuadr. 71/A, 280-290 cm | Charcoal | 3a | 1254±40 | 680 - 960 AD |
| 23 | Erl-3208 | sector E, Feature s..N., cuadr. 78/A, 270-280 cm | Charcoal | 3a | 1026±43 | 990 - 1160 AD |
| 24 | Erl-3204 | Unit 2B, Feature 113, cuadr. 30/117, 148 cm | Charcoal | 3a | 1261±37 | 680 - 950 AD |
| 25 | Erl-4792 | Unit 2A, Feature 121, cuadr. 29-30/101, 145 cm | Bone | 3a | 1275±37 | 680 - 890 AD |
| 26 | Erl-4783 | Unit 3, Feature 306, cuadr. 29/107, 75-80 cm | Charcoal | 3a | 1355±38 | 640 - 840 AD |
| 27 | Erl-4784 | Unit 3, Feature 300, cuadr. 28/108, 76 cm | Charcoal | 3a | 335±48 | 1460 - 1670 AD |
| 28 | Erl-5615 | Unit 9, Feature 963, cuadr. 5/A, 291 cm | Charcoal | 3a | 1238±53 | 680 - 980 AD |
| 29 | Erl-5616 | Unit 9, Feature 965, cuadr. 14/A, 300 cm | Charcoal | 3a | 1316±57 | 650 - 890 AD |
| 30 | Erl-3203 | Unit 1, Feature 46, cuadr. 25/B, 500 - 510 cm | carbonised Motacú-fruit* | 3b | 953±38 | 1030 - 1210 AD |
| 31 | Bln 5327 | Unit 1, Feature 39, cuadr. 25/B, 480 - 490 cm | Charcoal | 3b | 1134±33 | 890 - 1020 AD |
| 32 | Erl-3202 | Unit 1, Feature 37, cuadr. 32/A, 460 - 465 cm | Charcoal | 3b | 1186±66 | 690 - 1030 AD |
| 33 | Erl-3201 | Unit 1, Feature 36, cuadr. 30/B, 440 - 450 cm | Charcoal | 4a | 1043±43 | 980 - 1160 AD |
| 34 | Bln 5345 | Unit 1, Feature 26, cuadr. 27/A-B, 400 - 420 cm | Charcoal | 4a | 961±24 | 1030 - 1190 AD |
| 35 | Bln 5326 | Unit 1, Feature 26, cuadr. 27/A-B, 400 - 420 cm | Charcoal | 4a | 950±29 | 1040 - 1210 AD |
| 36 | Bln 5325 | Unit 1, Feature 25, cuadr. 26/A, 400 - 420 cm | Charcoal | 4a | 1077±28 | 900 - 1130 AD |
| 37 | Bln 5344 | Unit 1, Feature 25, cuadr. 26/A, 400 - 420 cm | Charcoal | 4a | 1036±39 | 990 - 1150 AD |
| 38 | Bln 5214 | Unit 1, Feature 25, cuadr. 25/B, 390-400 cm | Charcoal | 4a | 1065±29 | 980 - 1140 AD |
| 39 | Bln 5213 | Unit 1, Feature 26, cuadr. 25/B, 390-400 cm | Charcoal | 4a | 948±29 | 1040 - 1210 AD |
| 40 | Erl-3200 | Unit 1, Feature 22, cuadr. 29-30/A, 380 - 390 cm | Charcoal | 4a | 973±45 | 1020 - 1210 AD |
| 41 | Bln 5212 | Unit 1, Feature 19, cuadr. 25/B, 320-330 cm | Charcoal | 4a | 1050±31 | 980 - 1150 AD |
| 42 | Bln 5211 | Unit 1, Feature 17, cuadr. 30-31/A, 360-370 cm | Charcoal | 4a | 1030±28 | 990 - 1150 AD |
| 43 | Bln 5210 | Unit 1, Feature 50, cuadr. 23-25/A, 190-250 cm | Charcoal | 4b | 631±29 | 1300 - 1420 AD |
| 44 | Erl-3199 | Unit 1, Feature 101, cuadr. 22/A, 135-160 cm | Soot | 5 | 609±46 | 1300 - 1440 AD |
| 45 | Erl-4791 | Unit 1, Feature 4, cuadr. 29-30/A, 130-140 cm | Charcoal | 5 | 651±34 | 1290 - 1410 AD |
| 46 | KIA-40609 | Unit 9, Feature 904, cuadr. 18/A, 130-140 cm | Charcoal | 5 | 661±21 | 1300 - 1400 AD |

* Palm (*Attalea phalerata*)

The radiocarbon dates from the Mendoza site listed above have been previously published^12^.

# Supplementary Table 4 | Published radiocarbon dates from other Casarabe culture sites.

| **Lab-Code** | **Date BP** | **+/-** | **Provenience (Site, cut, level, etc.)** |
| --- | --- | --- | --- |
| SI-4044 | 2799 | 80 | Los Aceites, Level 6 (100-120 cm)^41^ |
| SI-4045 | 1664 | 80 | Los Aceites, Level 16 (300-320 cm)^41^ |
| SI-4048 | 764 | 60 | Palmasola, Cut 2, Level 9 (160-180 cm)^41^ |
| SI-4113 | 699 | 70 | Palmasola, Cut 5, Level 3 (50-70 cm)^41^ |
| SI-4114 | 664 | 60 | Mary, Level 3 (40-60 cm)^41^ |
| SI-4115 | 964 | 70 | Mary, Level 5 (80-100 cm)^41^ |
| SI-4116 | 994 | 80 | Mary, Level 12 (220-240 cm)^41^ |
| SI-4117 | 2784 | 160 | Mary, Level 15 (280-300 cm)^41^ |
| SI-4118 | 1384 | 60 | Mary, Level 17 (320-340 cm)^41^ |
| SI-4119 | 1729 | 75 | Mary, Level 19 (360-380 cm)^41^ |
| SI-4436 | 714 | 65 | Kiusíu, Level 2 (20-40 cm)^41^ |
| SI-4437 | 919 | 60 | Kiusíu, Level 5 (80-100 cm)^41^ |
| SI-4438 | 944 | 70 | Kiusíu, Level 8 (140-160 cm)^41^ |
| SI-4439 | 844 | 60 | Kiusíu, Level 9 (160-180 cm)^41^ |
| SI-4440 | 894 | 70 | Kiusíu, Level 13 (240-260 cm)^41^ |
| SI-4441 | 1444 | 60 | Kiusíu, Level 15 (280-300 cm)^41^ |
| SI-4442 | 1379 | 60 | Kiusíu, Level 17 (320-340 cm)^41^ |
| SI-5380 | 924 | 70 | Loma Alta de Casarabe, Cut 1, Level 3 (40-60 cm)^41^ |
| SI-5381 | 859 | 70 | Loma Alta de Casarabe, Cut 1, Level 6 (100-120 cm)^41^ |
| SI-5382 | 1084 | 65 | Loma Alta de Casarabe, Cut 1, Level 8 (140-160 cm)^41^ |
| SI-5383 | 854 | 70 | Loma Alta de Casarabe, Cut 1, Level 11 (200-220 cm)^41^ |
| SI-5870 | 1079 | 55 | Loma Alta de Casarabe, Cut 1, Level 16 (300-320 cm)^41^ |
| SI-5384 | 1164 | 90 | Loma Alta de Casarabe, Cut 1, Level 17 (320-340 cm^41^ |
| SI-5871 | 1424 | 75 | Loma Alta de Casarabe, Cut 1, Level 19 (360-380 cm)^41^ |
| SI-5872 | 1339 | 70 | Loma Alta de Casarabe, Cut 1, Level 26 (500-520 cm)^41^ |
| SI-5385 | 779 | 95 | Loma Alta de Casarabe, Cut 1, Level 31 (600-620 cm^41^ |
| SI-5873 | 704 | 120 | Loma Alta de Casarabe, Cut 1, Level 33 (640-660 cm)^41^ |
| SI-5386 | 1489 | 60 | Loma Alta de Casarabe, Cut 1, Level 38 (740-760 cm)^41^ |
| SI-5387 | 1639 | 70 | Loma Alta de Casarabe, Cut 1, Level 40 (780-800 cm)^41^ |
| SI-5875 | 1399 | 60 | Loma Alta de Casarabe, Cut 1, Level 41 (800-820 cm)^41^ |
| SI-5876 | 2709 | 145 | Loma Alta de Casarabe, Cut 1, Level 45 (890-900 cm)^41^ |
| SI-5393 | 1349 | 50 | Salvatierra, Cut 1, Level 3 (40-60 cm)^41^ |
| SI-5394 | 1129 | 80 | Salvatierra, Cut 1, Level 6 (100-120 cm)^41^ |
| SI-5390 | 1059 | 95 | Salvatierra, Cut 2, Level 6 (100-120 cm)^41^ |
| SI-5878 | 1064 | 45 | Salvatierra, Cut 2, Level 7 (120-140 cm)^41^ |
| SI-5391 | 1404 | 65 | Salvatierra, Cut 2, Level 8 (140-160 cm)^41^ |
| SI-5879 | 1029 | 50 | Salvatierra, Cut 2, Level 10 (180-200 cm)^41^ |
| SI-5392 | 844 | 70 | Salvatierra, Cut 2, Level 11 (200-220 cm)^41^ |
| none-1 | 2970 | 40 | Pancho Román; sample no. 5^43^ |
| none-2 | 1850 | 40 | Pancho Román; sample no. 6^43^ |
| none-3 | 920 | 40 | Pancho Román; unit 4; sample no. 12^43^ |
| none-4 | 111 | 40 | Pancho Román; unit 1; sample no. 13^43^ |
| none-5 | 690 | 40 | Pancho Román; unit 2; sample no. 16^43^ |
| none-6 | 900 | 40 | Pancho Román; unit 4; sample no. 22^43^ |
| none-7 | 970 | 40 | Pancho Román; unit 4; sample no. 23^43^ |
| none-8 | 940 | 40 | Pancho Román; unit 4; sample no. 24^43^ |
| none-9 | 1080 | 40 | Pancho Román; unit 4; sample no. 25^43^ |
| none-10 | 1110 | 40 | Pancho Román; unit 3; sample no. 28^43^ |
| none-11 | 1040 | 40 | Pancho Román; unit 3; sample no. 32^43^ |
| none-12 | 1200 | 40 | Pancho Román; unit 2; sample no. 49^43^ |

# Supplementary Table 5 | Criteria for the Monumental Mound Region settlement hierarchy.

Settlement Core Polygonal Qualitative characteristics

tier Area (ha) enclosure (ha)

1 22-12 314-147 diverse civic-ceremonial architecture

2-3 ranked polygonal enclosures

min. 7 straight causeways

height >15 m

2 6-2 41-21 diverse civic-ceremonial architecture

1 polygonal enclosure

3 0.5 2.5 single platform mound

4 0.3 n/a

# Supplementary Method│Oxcal code for the analysis of radiocarbon dates from Salvatierra.

Options()

{

PlusMinus=FALSE;

SD1=TRUE;

SD2=TRUE;

SD3=FALSE;

};

Plot()

{

Curve("SHCal20","shcal20.14c");

Sequence()

{

Boundary("Start 5");

Phase("5")

{

R_Date("KIA 31859", 1041, 20);

R_Date("MAMS 34445", 1033, 21);

R_Date("MAMS 48313", 1023, 22);

R_Date("MAMS 48315", 997, 24);

R_Date("MAMS 48312", 997, 23);

R_Date("MAMS 34447", 988, 22);

R_Date("MAMS 48323", 983, 25);

R_Date("MAMS 48311", 975, 23);

R_Date("KIA 38801", 957, 23);

R_Date("KIA 38807", 947, 21);

R_Date("KIA 40613", 876, 29);

R_Date("KIA 38811", 740, 23);

R_Date("KIA 38806", 596, 22);

};

Boundary("Transition 4/5");

Phase("4")

{

R_Date("Erl - 8016", 1278, 53);

R_Date("MAMS 48314", 1076, 24);

R_Date("KIA 38800", 1060, 25);

R_Date("Erl - 8012", 1044, 44);

R_Date("Erl - 8013", 1030, 44);

R_Date("KIA 38810", 1007, 22);

R_Date("MAMS 48331", 997, 24);

R_Date("MAMS 48321", 995, 25);

R_Date("MAMS 48335", 987, 26);

R_Date("MAMS 48334", 986, 22);

R_Date("Erl - 8015", 985, 45);

R_Date("MAMS 34449", 982, 23);

R_Date("MAMS 34450", 981, 22);

R_Date("MAMS 48320", 979, 25);

R_Date("MAMS 48333", 975, 27);

R_Date("MAMS 48322", 967, 25);

R_Date("KIA 31857", 956, 25);

R_Date("MAMS 48318", 951, 23);

R_Date("KIA 32717", 948, 25);

R_Date("MAMS 48316", 938, 22);

R_Date("KIA 38809", 932, 22);

R_Date("MAMS 48348", 929, 23);

R_Date("MAMS 48317", 910, 23);

R_Date("MAMS 48319", 904, 25);

R_Date("MAMS 48345", 904, 23);

R_Date("KIA 38814", 896, 32);

};

Boundary("Transition 3/4");

Phase("3")

{

R_Date("MAMS 48328", 1413, 27);

R_Date("MAMS 48304", 1287, 23);

R_Date("MAMS 48306", 1286, 24);

R_Date("MAMS 48307", 1286, 22);

R_Date("MAMS 48327", 1282, 27);

R_Date("Erl - 8014", 1275, 46);

R_Date("MAMS 48344", 1246, 23);

R_Date("KIA 38802", 1240, 28);

R_Date("MAMS 48336", 1240, 24);

R_Date("KIA 32718", 1239, 31);

R_Date("KIA 38813", 1228, 23);

R_Date("KIA 38812", 1223, 26);

R_Date("MAMS 34453", 1204, 23);

R_Date("KIA 32719", 1201, 24);

R_Date("MAMS 48332", 1188, 21);

R_Date("MAMS 48337", 1184, 24);

R_Date("MAMS 48342", 1164, 23);

R_Date("MAMS 48343", 1160, 22);

R_Date("KIA 31858", 1156, 25);

R_Date("MAMS 34446", 1149, 22);

R_Date("MAMS 34451", 1145, 22);

};

Boundary("Transition 2/3");

Phase("2")

{

R_Date("MAMS 48347", 1412, 23);

R_Date("MAMS 48346", 1377, 23);

R_Date("MAMS 48310", 1357, 26);

R_Date("MAMS 48325", 1351, 25);

R_Date("KIA 31854", 1350, 20);

R_Date("KIA 31855", 1341, 24);

R_Date("Bln - 5860", 1337, 39);

R_Date("KIA 38804", 1335, 26);

R_Date("MAMS 48324", 1322, 25);

R_Date("MAMS 48308", 1321, 26);

R_Date("KIA 38803", 1316, 27);

R_Date("Bln 5861", 1311, 26);

R_Date("MAMS 48309", 1304, 25);

R_Date("KIA 38805", 1300, 23);

R_Date("Bln-5947 LI", 1275, 39);

R_Date("MAMS 48303", 1251, 23);

R_Date("KIA 38808", 1224, 24);

};

Boundary("Transition 1/2");

Phase("1")

{

R_Date("MAMS 31856", 1623, 30);

R_Date("MAMS 34454", 1589, 22);

R_Date("KIA 40614", 1542, 21);

R_Date("MAMS 48340", 1494, 24);

R_Date("KIA 32720", 1489, 27);

R_Date("MAMS 48326", 1481, 26);

R_Date("MAMS 48339", 1475, 25);

R_Date("MAMS 48341", 1475, 23);

R_Date("MAMS 48329", 1467, 27);

R_Date("MAMS 34448", 1464, 22);

R_Date("MAMS 48338", 1445, 27);

R_Date("MAMS 48330", 1441, 22);

R_Date("Bln-5945 L", 1389, 30);

};

Boundary("End 1");

};

};

Boundary("Transition 0/1");

Phase("0")

{

R_Date("Erl 8011", 2594, 49);

R_Date("MAMS 48305", 2995, 25);

R_Date("Bln-5946 LI", 3319, 42);

};

Boundary("End 1");

};

};
