# Supplementary material for: Lidar reveals pre-Hispanic low-density urbanism in the Bolivian Amazon
Source: Nature. 2022 May 25;606(7913):325–8. doi: 10.1038/s41586-022-04780-4 (PMC9177426; doi:10.1038/s41586-022-04780-4)
Supplement: Supplementary file 2 — Reporting Summary [file 41586_2022_4780_MOESM2_ESM.pdf]

nature portfolio

Corresponding author(s): Heiko Prümers

Last updated by author(s): Mar 4, 2022

Reporting Summary

Nature Portfolio wishes to improve the reproducibility of the work that we publish. This form provides structure for consistency and transparency in reporting. For further information on Nature Portfolio policies, see our Editorial Policies and the Editorial Policy Checklist.

Statistics

For all statistical analyses, confirm that the following items are present in the figure legend, table legend, main text, or Methods section.

n/a	Confirmed	
☐	☒	The exact sample size (n) for each experimental group/condition, given as a discrete number and unit of measurement
☐	☒	A statement on whether measurements were taken from distinct samples or whether the same sample was measured repeatedly
☐	☒	The statistical test(s) used AND whether they are one- or two-sided<br>*Only common tests should be described solely by name; describe more complex techniques in the Methods section.*
☒	☐	A description of all covariates tested
☒	☐	A description of any assumptions or corrections, such as tests of normality and adjustment for multiple comparisons
☐	☒	A full description of the statistical parameters including central tendency (e.g. means) or other basic estimates (e.g. regression coefficient) AND variation (e.g. standard deviation) or associated estimates of uncertainty (e.g. confidence intervals)
☒	☐	For null hypothesis testing, the test statistic (e.g. F, t, r) with confidence intervals, effect sizes, degrees of freedom and P value noted<br>*Give P values as exact values whenever suitable.*
☒	☐	For Bayesian analysis, information on the choice of priors and Markov chain Monte Carlo settings
☒	☐	For hierarchical and complex designs, identification of the appropriate level for tests and full reporting of outcomes
☒	☐	Estimates of effect sizes (e.g. Cohen's d, Pearson's r), indicating how they were calculated

Our web collection on statistics for biologists contains articles on many of the points above.

Software and code

Policy information about availability of computer code

Data collection	RIEGL RiAcquire
Data analysis	RIEGL RiAnalyze, ARCGis

For manuscripts utilizing custom algorithms or software that are central to the research but not yet described in published literature, software must be made available to editors and reviewers. We strongly encourage code deposition in a community repository (e.g. GitHub). See the Nature Portfolio guidelines for submitting code & software for further information.

Data

Policy information about availability of data

All manuscripts must include a data availability statement. This statement should provide the following information, where applicable:

- Accession codes, unique identifiers, or web links for publicly available datasets
- A description of any restrictions on data availability
- For clinical datasets or third party data, please ensure that the statement adheres to our policy

All relevant data, code, and materials are provided with the paper.

Field-specific reporting

Please select the one below that is the best fit for your research. If you are not sure, read the appropriate sections before making your selection.

☐ Life sciences ☒ Behavioural & social sciences ☐ Ecological, evolutionary & environmental sciences

For a reference copy of the document with all sections, see nature.com/documents/nr-reporting-summary-flat.pdf

Behavioural & social sciences study design

All studies must disclose on these points even when the disclosure is negative.

Study description	Spatial analysis of lidar data, quantitative
Research sample	Laser mapping of six transects (10-85 km2) totalling 204 km2
Sampling strategy	Targeted sampling, areas with know concentrations of archaeological sites
Data collection	The sensor used to collect lidar data was a Riegl VUX-1 scanner, with a Trimble APX-15 UAV GNSS, attached to a Eurocopter AS350 helicopter using a custom mount. Laser Pulse Repetition Rate (PRR) was 200 kHz. Flight altitude was 200 m above ground 284 level, airspeed was 45 knots. Missions were flown in 200 m parallel strips, with 50% overlap.
Timing	There was no gap between collecting periods.
Data exclusions	n/a
Non-participation	n/a
Randomization	n/a

Reporting for specific materials, systems and methods

We require information from authors about some types of materials, experimental systems and methods used in many studies. Here, indicate whether each material, system or method listed is relevant to your study. If you are not sure if a list item applies to your research, read the appropriate section before selecting a response.

Materials & experimental systems

n/a	Involved in the study	
☒	☐	Antibodies
☒	☐	Eukaryotic cell lines
☐	☒	Palaeontology and archaeology
☒	☐	Animals and other organisms
☒	☐	Human research participants
☒	☐	Clinical data
☒	☐	Dual use research of concern

Methods

n/a	Involved in the study	
☒	☐	ChIP-seq
☒	☐	Flow cytometry
☒	☐	MRI-based neuroimaging

Palaeontology and Archaeology

Specimen provenance	Permit to collect lidar data was obtained from the Departmental Autonomous Government of Beni and of the Bolivian Ministry of Cultures and Tourism, Bolivia.
Specimen deposition	n/a
Dating methods	Radiocarbon essay are reported from previous excavations in the area by the authors and colleagues.

☒ Tick this box to confirm that the raw and calibrated dates are available in the paper or in Supplementary Information.

Ethics oversight	No ethical approval is necessary to conduct lidar mapping in rural areas of Bolivia.

Note that full information on the approval of the study protocol must also be provided in the manuscript.
